# The evolutionary consequences of human–wildlife conflict in cities

**DOI:** 10.1111/eva.13131

**Published:** 2020-09-29

**Authors:** Christopher J. Schell, Lauren A. Stanton, Julie K. Young, Lisa M. Angeloni, Joanna E. Lambert, Stewart W. Breck, Maureen H. Murray

**Affiliations:** ^1^ School of Interdisciplinary Arts and Sciences University of Washington Tacoma Tacoma WA USA; ^2^ Department of Zoology and Physiology University of Wyoming Laramie WY USA; ^3^ Program in Ecology University of Wyoming Laramie WY USA; ^4^ USDA‐WS‐National Wildlife Research Center‐Predator Research Facility Millville UT USA; ^5^ Department of Biology Colorado State University Fort Collins CO USA; ^6^ Program in Environmental Studies and Department of Ecology and Evolutionary Biology University of Colorado‐Boulder Boulder CO USA; ^7^ USDA‐WS‐National Wildlife Research Center Fort Collins CO USA; ^8^ Department of Fish, Wildlife, and Conservation Biology Fort Collins CO USA; ^9^ Urban Wildlife Institute and Davee Center for Epidemiology and Endocrinology Chicago IL USA

**Keywords:** adaptive management, genetic, human–wildlife conflict, phenotypic plasticity, social learning, urban evolution

## Abstract

Human–wildlife interactions, including human–wildlife conflict, are increasingly common as expanding urbanization worldwide creates more opportunities for people to encounter wildlife. Wildlife–vehicle collisions, zoonotic disease transmission, property damage, and physical attacks to people or their pets have negative consequences for both people and wildlife, underscoring the need for comprehensive strategies that mitigate and prevent conflict altogether. Management techniques often aim to deter, relocate, or remove individual organisms, all of which may present a significant selective force in both urban and nonurban systems. Management‐induced selection may significantly affect the adaptive or nonadaptive evolutionary processes of urban populations, yet few studies explicate the links among conflict, wildlife management, and urban evolution. Moreover, the intensity of conflict management can vary considerably by taxon, public perception, policy, religious and cultural beliefs, and geographic region, which underscores the complexity of developing flexible tools to reduce conflict. Here, we present a cross‐disciplinary perspective that integrates human–wildlife conflict, wildlife management, and urban evolution to address how social–ecological processes drive wildlife adaptation in cities. We emphasize that variance in implemented management actions shapes the strength and rate of phenotypic and evolutionary change. We also consider how specific management strategies either promote genetic or plastic changes, and how leveraging those biological inferences could help optimize management actions while minimizing conflict. Investigating human–wildlife conflict as an evolutionary phenomenon may provide insights into how conflict arises and how management plays a critical role in shaping urban wildlife phenotypes.

## 1 INTRODUCTION

The rapid expansion of urban areas worldwide is markedly increasing the frequency of encounters humans have with wildlife (Soulsbury & White, 2015). Though most encounters are positive or neutral (Soga & Gaston, 2020), encounters can result in negative outcomes (i.e., conflict) that include property loss or damage, pet loss, disease transmission, physical injury, and human or wildlife fatalities (Richardson et al., 2020; Treves et al., 2006). Human–wildlife conflict has been extensively studied, emphasizing the drivers, consequences, and associated mitigation strategies to resolve emerging conflicts. Human attitudes toward wildlife (Dickman, 2010; Dickman et al., 2013), human activities and behaviors (Penteriani et al., 2016), wildlife adaptation and exploitation of anthropogenic resources (Ditchkoff et al., 2006; Honda et al., 2018; Kumar et al., 2019), and climate‐driven biotic redistributions (Pecl et al., 2017) all contribute to the spatial and temporal distribution of conflict. Coupled with urbanization and climate‐induced environmental changes, the spatiotemporal extent and magnitude of conflict is increasing, with organisms under intensifying selective pressures (Donihue & Lambert, 2014; Johnson & Munshi‐South, 2017; Turner et al., 2018). Moreover, conflicts have substantial financial costs, resulting in nearly $230 million (USD) in compensation across 50 countries since 1980 (Ravenelle & Nyhus, 2017). Hence, one of the most urgent conservation and management priorities of this century is developing adaptive management strategies that integrate social, biological, and temporal variables to mitigate, resolve, and prevent conflicts (Dickman, 2010; Ives & Kendal, 2014; Jørgensen et al., 2019).

**FIGURE 1:**
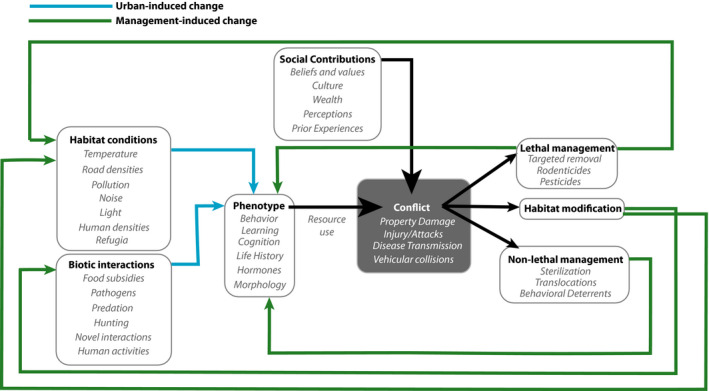
Conceptual framework illustrating the processes contributing to shaping phenotypes, human–wildlife conflict, and resulting management actions in urban systems. Habitat conditions and biotic interactions combine to produce both adaptive (i.e., natural and sexual selection) and nonadaptive (i.e., reduce gene flow, genetic drift) evolutionary changes that affect use of limited resources by urban organisms. Varying social attributes of a city, including religion, socioeconomics, political, and cultural perspectives, coalesce with urban organismal adaptation to shape human–wildlife conflict (black lines). The magnitude, severity, and frequency of those conflicts then inform the type of management decisions and actions implemented, and those actions produce evolutionary feedback mechanisms that continually refine urban phenotypes. Hence, phenotypic changes occur due to urban landscape conditions (blue lines) and management actions (green lines)

Prior work detailing adaptive wildlife management frameworks emphasizes the need for evidence‐based research that incorporates the inherent social–ecological nature of human–wildlife conflict to improve management decisions (Enck et al., 2006; Richardson et al., 2020). Adaptive impact management programs (AIM, also referred to as adaptive social impact management) are built on the assumption that change is inevitable, requiring programmatic flexibility to adapt to social, cultural, and biological shifts over time (Gregory et al., 2006; Ives & Kendal, 2014; Kaplan‐Hallam & Bennett, 2018). Both adaptive management and evolutionary biology are thus founded on an understanding of change over time (Lambert & Donihue, 2020). Moreover, management optimization is itself a selective pressure; management decisions impact population abundance and demography, and deter behaviors that may exacerbate conflict with people (Barrett et al., 2019; Jørgensen et al., 2019; Swan et al., 2017). As a result, management can operate as a selective force that shapes—and is shaped by—wildlife responses (Figure 1), yet evolutionary processes are rarely integrated into AIM frameworks explicitly.

Interactions between humans and wildlife, including competition and conflict, are not new to human history. Indeed, human commensals and domesticated species have coevolved with human societies over thousands of years, documented as far back as the Pleistocene and Holocene (Clucas & Marzluff, 2011; Hendry et al., 2017; Hulme‐Beaman et al., 2016; Sullivan et al., 2017). Human behavior has had substantial evolutionary effects with measurable shifts in morphology, abundances, and community interactions (Erlandson & Rick, 2010; Kemp et al., 2020; Sullivan et al., 2017). More recently, selective breeding, removal, and hunting have acted as strong selective agents driving directional, stabilizing, or disruptive selection that shapes the evolutionary trajectories of organisms inhabiting anthropogenic habitats (Hendry et al., 2017). Relative to historical patterns of interactions among commensals and humans, selective pressures in modern cities are orders of magnitude greater due to concentrated anthropogenic drivers across space and time. Anthropogenic landscape conversion (e.g., vegetation cover and diversity, waste and pollution systems, transportation infrastructure) and human activities (e.g., lethal removal, proliferation of domestic species, recreational use of green space) compound to create strong selective agents that establish individual trait‐based and species filtering (Alberti, 2015; Ellwanger & Lambert, 2018; Ouyang et al., 2018; Pagani‐Núñez et al., 2019). Moreover, the dynamics of policy, governance, market fluctuations, and zoning practices generate substantial—and uniquely urban—spatiotemporal heterogeneity over relatively small scales (Liu et al., 2007; Pataki, 2015; Pickett et al., 2016). For these reasons, the convergence of human–wildlife conflict, adaptive impact management, and urban evolution provide an exceptional opportunity to articulate a framework incorporating evolving biotic interactions as key for wildlife management.

We provide a transdisciplinary synthesis that integrates principles from human–wildlife conflict and urban evolutionary ecology to illustrate that conflict and management decisions are both a signal of selection and a selective agent that directly affect evolutionary change in urban populations (Figure 1). First, we review the ecological drivers of urban conflict globally. Second, we explain how sociocultural factors underpin conflict and vary tremendously across scales (e.g., neighborhood, township, census block, city level). Third, we emphasize how management decisions in response to conflict work to select and reinforce specific wildlife traits over others. Lastly, we discuss how urban evolutionary biology can provide a toolkit to help optimize adaptive wildlife management strategies. We concurrently emphasize that high variability in urban metrics across gradients of developed and developing cities—particularly their structural, abiotic, and biotic components (Moll et al., 2019), as well as their developmental histories and trajectories—dictates the implementation and success of management strategies. We define *urban* according to the dynamic and nuanced definition articulated by Moll et al. (2019), in which the relative proportion of gray space land cover (e.g., buildings, impervious surfaces) to green and blue structural components (e.g., parks, waterways) is high over space and time.

Our framework builds on previous syntheses (Jørgensen et al., 2019; Nyhus, 2016; Swan et al., 2017) by explaining how evolutionary concepts can be harnessed to develop broad management approaches that ameliorate conflict and promote human–wildlife coexistence in urban areas globally (Cook & Sgrò, 2018).

## 2 ECOLOGICAL DRIVERS OF CONFLICT AND ASSOCIATED BIOLOGICAL OUTCOMES

**FIGURE 2:**
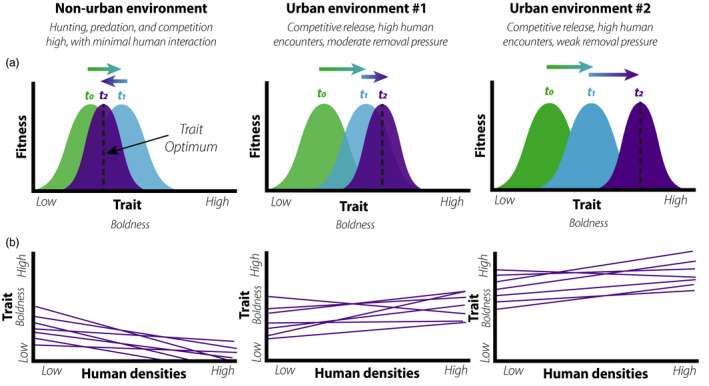
Niche differentiation and variance in selective modes, strength, and behavioral trait plasticity in response to human–animal interactions. (a) In nonurban environments, stabilizing selection over time favors low‐to‐moderate boldness with bolder individuals hunted or lost to predation. Conversely, in urban environments competitive release and decreased hunting promotes directional selection toward bolder phenotypes. However, between‐city variance in the intensity of management action (e.g., removal pressure) can induce mean‐level phenotypic variance in traits. (b) Reaction norms toward anthropogenic factors (e.g., human densities, human presence) are shaped by human–animal interactions. Though individual plasticity persists in all environments (purple lines) with similar directionality, mean‐level population differences in boldness emerge due to differences in the type and frequency of human encounters across urban and nonurban environments, and between cities

**FIGURE 3:**
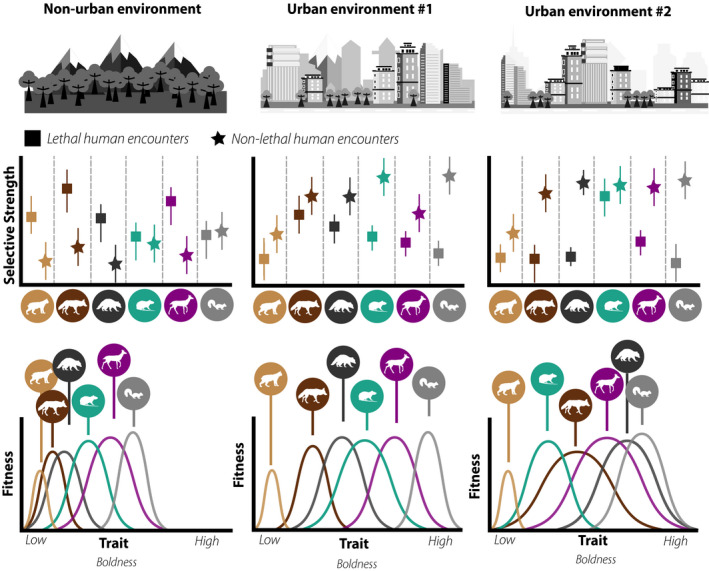
Theoretical predictions of illustrating differences in performance curves, fitness, and trait variance of urban wildlife as a function of habitat conditions and human–animal interactions. (a) Variance in the ratio of positive, neutral, or negative human–wildlife interactions (i.e., lethal vs. nonlethal human encounters) creates unique selective gradients across species, in which the degree of lethal to nonlethal human encounters promotes specific performance curves for behaviors such as boldness (b). The overall number of nonlethal human interactions substantially increases in cities, greatly contributing to urban versus nonurban differences in behavioral phenotypes. A higher proportion of lethal relative to nonlethal human encounters selects for shy phenotypes generally across all wildlife. Species differences persist due to variance in social perceptions, conflict frequency, and conflict severity of varying wildlife taxa. Increasing the relative separation between lethal and nonlethal interactions may additionally contribute to increasing phenotypic plasticity, in which large differentials between the two types of interactions allow for a larger variety of phenotypes to persist in the population. For instance, coyotes and deer in urban environment #2 have substantially more nonlethal human encounters with minimal risk of lethal interactions as compared to urban environment #1. The performance curves for those species are thus wider in city #2. Between‐city differences in phenotypic signatures may be the result of selection, developmental experiences, and/or learning the sources of rewards. Error bars denote individual variance in human experiences across a theoretical population. Selected mammals in the figure are those commonly found in North American cities, including (from left to right) the following: bobcats, *Lynx rufus*; coyotes, *Canis latrans*; raccoons, *Procyon lotor*; brown rats, *Rattus norvegicus*; white‐tailed deer, *Odocoileus virginianus*; and eastern gray squirrels, *Sciurus carolinensis*

The combination of human‐induced habitat changes and novel biotic interactions produces divergent fitness landscapes that promote specific phenotypic traits in cities (Alberti et al., 2017; Ouyang et al., 2018). Urban wildlife exhibit increased nocturnality (Gaynor et al., 2018), cognitive and problem‐solving innovations (Audet et al., 2016; Snell‐Rood & Wick, 2013), heightened tolerance and habituation (Lowry et al., 2013; Sol et al., 2013), and dietary niche shifts (Murray, Lankau, et al., 2020; Pagani‐Núñez et al., 2019), all of which facilitate survival and reproductive success in cities. Phenotypic shifts and plasticity in urban contexts can promote local adaptation by reducing the likelihood of human–wildlife encounters (Ditchkoff et al., 2006; Tuomainen & Candolin, 2011). However, in some instances local adaptation may increase the likelihood of human–wildlife encounters (Soulsbury & White, 2015), occasionally resulting in contentious interactions that reduce organismal fitness due to lethal removal actions (Honda et al., 2018). In addition, detecting phenotypic signals of local adaptation varies considerably by species (Santini et al., 2019) and city scale (Strubbe et al., 2020), in which variance in life histories and niche requirements establish trait‐reaction norms for individuals and species (Tuomainen & Candolin, 2011). Variance in environmental conditions and management actions within and across cities can further result in niche differentiation of adjacent populations that explain the origins of trait adaptations to human‐dominated landscapes (Figures 2 and 3).

Investigating the pathways by which human‐driven ecological conditions shape adaptation and conflict will help illuminate how wildlife management influences evolutionary outcomes of urban wildlife. Those pathways can operate either at the landscape level (i.e., anthropogenic habitat conditions) or at the community level (i.e., biotic interactions) with projections to the organismal level that affect population growth and abundance in cities (Figure 1). In addition, phenotypic changes in response to conflict‐inducing environmental factors can be adaptive, nonadaptive, or maladaptive (Brady & Richardson, 2017; Derry et al., 2019).

### 2.1 Road densities and vehicle collisions

Wildlife–vehicle collisions are one of the most prominent conflicts resulting in restricted animal movement and mortality, especially when roads fragment contiguous habitats (Balkenhol & Waits, 2009; Brady & Richardson, 2017; LaPoint et al., 2015). Roads are nearly ubiquitous in developed landscapes, and represent a major source of wildlife fatalities, property damage, and in many instances human injury and mortality (Brady & Richardson, 2017; Proppe et al., 2017). Heightened road densities in urban environments present a salient environmental challenge that can restrict successful colonization of viable urban habitats. Though taxa from multiple clades are affected, mortality risks are especially high for large vertebrates within cities (Edelhoff et al., 2020; Honda et al., 2018; Johnson et al., 2020) and at the urban–wildland interface (Proctor et al., 2020; St. Clair et al., 2019; Wynn‐Grant et al., 2018), where human‐modified attributes of the landscape and speed limits increase (Neumann et al., 2012). All these factors contribute to the reduced occupancy and population abundances of larger fauna in urban systems. Moreover, there is a rich and recent literature that suggests road densities in urban systems reduce gene flow and operate as genetic bottlenecks for an array of taxa (Kozakiewicz et al., 2019; Riley et al., 2006; Trumbo et al., 2019), highlighting the salience of roads as drivers of adaptive and nonadaptive evolutionary change (Brady & Richardson, 2017).

To circumnavigate this challenge, wildlife passages are installed over and under roads (Riley et al., 2014) and wildlife populations increase their nocturnal activity as a means of avoiding periods of high human activity and vehicle traffic volume (Baker et al., 2007; Murray & St. Clair, 2015). Evidence across passerines additionally suggests natural selection can occur for morphological changes to wing and body size that reduce vehicle collisions (Brown & Bomberger Brown, 2013; Santos et al., 2016). In urban mammals, high mortality rates due to vehicle collisions may drive an increase in body size, litter size, and faster maturation (Santini et al., 2019), suggesting that road densities may serve to alter pace‐of‐life syndromes. Further, increased disturbances (e.g., road noise and anthropogenic light at night) and pollutants (e.g., heavy metals, chemical contaminants) associated with high road densities may induce adaptive genetic change or drive mutagenic effects that produce detrimental changes in genes (Brady & Richardson, 2017). The pace and spatial scale of these changes can range considerably with road densities and proximity; however, recent work in large fauna with large dispersal ranges and slow paces of life suggests rapid signals of evolution at small spatial scales (Adducci et al., 2020; DeCandia et al., 2019; Richardson et al., 2014; Schell, 2018). Determining the scale and rate of evolutionary change due to road ecology will be necessary for adaptively mitigating conflicts as they arise (Brady & Richardson, 2017).

### 2.2 Property damage and infrastructure

The built environment can create compounding mortality risks for wildlife in two distinct ways. The first risk involves structures themselves as threats to wildlife survival. For instance, multistory commercial and industrial buildings with highly reflective windows pose a significant threat to birds, especially males and juveniles, via window strikes (Hager et al., 2013; Kahle et al., 2016; Loss et al., 2014). A second type of mortality risk, property damage caused by wildlife, triggers targeted management actions often resulting in lethal control actions to remove selected individuals (McCleery et al., 2014; Swan et al., 2017). Various taxa damage commercial and residential properties by using structures for refugia (Murray et al., 2018; VerCauteren et al., 2010), whereas defacement of other properties via wildlife‐generated fecal waste decreases aesthetic value of the property (Soulsbury & White, 2015). Retaliatory killing and extirpation techniques used to alleviate such conflicts likely place a significant selective pressure on target wildlife involved in associated disturbances (Swan et al., 2017).

### 2.3 Food provisioning

Although consumption of anthropogenic food resources is not a prerequisite of urban living (Newsome et al., 2015; Stillfried, Fickel, et al., 2017), cities likely favor species that learn to capitalize on human subsidies and refuse (Oro et al., 2013). Food provisioning of wildlife is a major source of conflict in cities (Dubois & Fraser, 2013) because animals that learn to associate humans with food may approach humans, residencies, and vehicles seeking food, increasing the likelihood of disease transmission, injury, or mortality (Cox & Gaston, 2018; Murray, Becker, et al., 2016; Oro et al., 2013; Sorensen et al., 2014; Strandin et al., 2018). Food provisioning may be especially problematic when (a) dependency on humans for food results in a decrease in natural behaviors and a more docile or tame phenotype (Geffroy et al., 2015; Lamb et al., 2017; St. Clair et al., 2019), or (b) habituation and increased boldness leads to a more aggressive phenotype (Cox & Gaston, 2018; Dubois & Fraser, 2013; Kumar et al., 2019). Scrounging and kleptoparasitism (i.e., stealing of food) by wildlife is common in cities (Beisner et al., 2015; Brotcorne et al., 2017; Goumas et al., 2019) and may drive advanced cognitive abilities and innovations that enable food acquisition from manufactured structures such as bottles and garbage bins (Arbilly et al., 2014; Ducatez et al., 2017; Griffin et al., 2017; Morand‐Ferron et al., 2007).

Reliable resources in cities may also alter wildlife movement patterns with important implications for conflict (Lowry et al., 2013; Wong & Candolin, 2015). Cities offer a relatively stable source of food from garbage, provisioned food, and cultivated plants and access to water (Cox & Gaston, 2018). In some instances, wildlife venture into urbanized areas to access more abundant natural resources and avoid competition or predation from other organisms deterred by higher human activity (Moll et al., 2018; Stillfried, Gras, Börner, et al., 2017; Stillfried, Gras, Busch, et al., 2017, 2017, 2017). The spatial distribution of food subsidies restructures species interactions and shapes the relative distribution of native versus non‐native species (Dorresteijn et al., 2015; Fischer et al., 2012), as non‐native species' ability to exploit resources and colonize urban habitats inhibits future colonization events of native species (i.e., priority effects; Lepczyk, Aronson, et al., 2017; Shochat et al., 2010; Urban & De Meester, 2009). Further, access to these stable resources helps explain why wildlife populations around the world are abandoning migration (Møller et al., 2014; Wilcove & Wikelski, 2008), often contributing to property damage in parks, aggressive encounters, and vehicular collisions (Dolbeer et al., 2014; Found & St. Clair, 2019; Hubbard & Nielsen, 2009).

Finally, direct effects of food provisioning on individuals, such as increased body mass and altered mating strategies, can have cascading effects on populations, communities, and ecosystems (Cox & Gaston, 2018; Oro et al., 2013). Bird feeding in particular has been linked to increased survival, advancement of breeding, and increased likelihood of pathogen transmission (Robb et al., 2008). Further, intentional use of bird feeders may result in unintentional and unwanted feeding of other omnivorous species. Processed foods are typically high in sugar, salt, and fat and low in protein, leading to hyperglycemia (Schulte‐Hostedde et al., 2018), and decomposing food can lead to harmful increased exposure to toxins from fungal metabolites (Murray, Hill, et al., 2016). Recent evidence linking human‐associated foods to genes for metabolism of high fat and starch (Harris & Munshi‐South, 2017; Ravinet et al., 2018), as well as physiological and microbiome adaptations in house sparrows (Gadau et al., 2019; Teyssier et al., 2018), provides emerging evidence that food subsidies can lead to the adaptive evolution of novel traits (Rivkin et al., 2019).

### 2.4 Domestic pets and human activities

The proliferation of domestic and feral pets disrupts trophic structure through predation, disease transmission, and general wildlife disturbance (Nyhus, 2016). Outdoor domestic cats (*Felis catus*) are a significant threat to bird and rodent populations in urban areas (Cove et al., 2018; Kays et al., 2020; Lepczyk, La Sorte, et al., 2017), and also present a major driver of conflict with other urban carnivores (Gehrt et al., 2013; Kays et al., 2015). In addition, outdoor cats are often reservoirs for the spread of several diseases including leptospirosis and toxoplasmosis that are transmissible to humans and other pets (Chalkowski et al., 2019; Dabritz & Conrad, 2010; Schuller et al., 2015). Domestic dogs (*Canis lupus familiaris*) are similarly a major driver of conflict, with wild predators such as coyotes (*Canis latrans*) and leopards (*Panthera pardus*) killing domestic dogs in cities, leading to emotional and economic trauma (Butler et al., 2015; Hughes & Macdonald, 2013) or, alternatively, positive benefits such as reduced rabies risk to humans (Braczkowski et al., 2018). Domestic dogs also increase the probability of human–carnivore conflict in green spaces (Penteriani et al., 2016) and built environments across the globe (Bhatia et al., 2013; Braczkowski et al., 2018; Butler et al., 2015; Hughes & Macdonald, 2013).

Human activities and recreation also directly play a role in eliciting conflicts. Recent work suggests that human presence results in a landscape of fear, which dictates daily activity budgets and spatiotemporal use of habitat by wildlife (Clinchy et al., 2016; Nickel et al., 2020; Suraci et al., 2019). The effect of humans persists for species even on the urban–wildland boundary, suggesting that mere human presence is strong enough to drive behavioral strategies that reduce human–wildlife encounters. For mammalian carnivores in particular, human activity can dissolve spatial and temporal avoidance of heterospecific competitors as a means of avoiding human encounters (Smith et al., 2017, 2018). Successful avoidance, however, is often compromised as human recreational trails in urban areas increasingly reduce refuges by fragmenting natural remnants (Ballantyne et al., 2014).

### 2.5 Health and disease

Urban living can also promote human–wildlife conflict arising from wildlife disease (Murray et al., 2019). Some wildlife pathogens such as canine distemper or rabies can directly cause changes in wildlife behavior that promote conflict. For example, raccoons (*Procyon lotor*) infected with canine distemper virus commonly exhibit abnormal behavior including lethargy, ataxia, and less wariness toward humans (Cranfield et al., 1984). Similarly, carnivores infected with the rabies virus typically exhibit increased aggression (Wang et al., 2010). Removal of infected individuals may impose a selective pressure favoring pathogen resistance. However, such infections are less likely to lead to selective removal if infected individuals cannot be readily identified based on behavior or appearance. Instead, conflict may arise due to human perception of public health risks from zoonotic pathogens transmissible to humans and consequently lower tolerance for wildlife presence. For example, urban coyote populations can have rates of tapeworm (*Echinococcus locularis*) infections as high as 65% (Luong et al., 2020), prompting public concern regarding exposure to parasites in urban green spaces (Deplazes et al., 2004).

Among the most profound examples of human–wildlife disease transmission is the current global COVID‐19 pandemic that is severely affecting public health, society, and the world economy (Chakraborty & Maity, 2020; Messmer, 2020). Evidence suggests bats are a natural reservoir host for the novel coronavirus, SARS‐CoV‐2 (Boni et al., 2020; MacFarlane & Rocha, 2020). Continued urbanization and its resulting expansion of human activities directed at wildlife (e.g., wildlife markets) and use of urban structures by wildlife (e.g., highway underpasses, culverts, buildings) have facilitated increased human–bat urban interactions around the world (Li & Wilkins, 2014; Russo & Ancillotto, 2015). At the same time, natural roosting areas outside of urban areas (e.g., forests, caves) have been reduced due to human activity (e.g., logging, agriculture, guano harvesting, limestone quarrying), likely facilitating the increased activity and use of urban areas (Russo & Ancillotto, 2015). The contexts that promote pathogen spillover between wildlife and humans (i.e., close contact between multiple species, compounding stressors that may increase infection susceptibility) are expected to increase with urbanization unless we manage habitat to allow wildlife persistence without coming in close contact with people (Messmer, 2020; Murray et al., 2019). In addition, human–human transmission from disease spillover events versus zoonoses reliant on transmission from wildlife (e.g., leptospirosis, rabies, Lyme disease) may require different management and public health responses that mitigate the impacts of disease spread.

## 3 SOCIOCULTURAL DETERMINANTS OF CONFLICT

**FIGURE 4:**
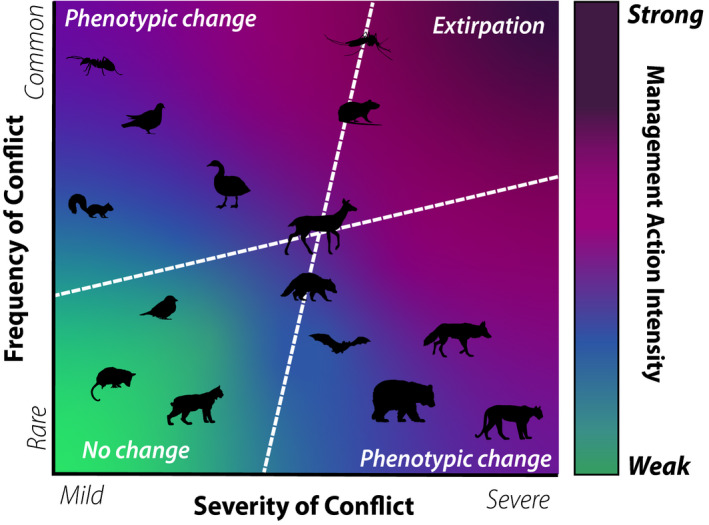
Frequency and severity of conflicts drive management action intensity and shape evolutionary trajectories of urban wildlife. The frequency and severity of conflicts dictate the strength of management action placed on wildlife, with considerable variability across taxa. Phenotypic change is predicted when frequency, severity, or both are particularly high. In instances where conflict severity and frequency are benign or mild, human–wildlife conflict is unlikely to induce evolutionary change (bottom‐left quadrant). Extreme severity and conflict, however, may lead to extirpation from an urban habitat (top‐right quadrant) or prevent urban colonization. In addition, conflict with larger fauna may be graded as more severe, though infrequent

Cost assessment of conflict is substantially modulated by how humans perceive conflict‐causing species (Dickman, 2010; Soulsbury & White, 2015). Human perceptions of organisms as either benign or malignant can consciously and unconsciously drive how we respond to emergent conflicts from target species (Kaplan‐Hallam & Bennett, 2018). Heterogeneity in the social, cultural, economic, and personal attributes of society contributes to shaping individual human beliefs and values of wildlife (Ives & Kendal, 2014; Manfredo & Dayer, 2004), subsequently informing the type and strength of management strategies implemented (Figure 4). How conflict‐causing species are managed is thus inherently social, with cascading evolutionary consequences for the target species. As organisms navigate various neighborhoods in cities, they likely encounter people across jurisdictional boundaries and municipalities with different beliefs, attitudes, and policies for managing the target species (Draheim et al., 2019; Enck et al., 2006; Manfredo et al., 2020). Reciprocally, variation in the frequency, severity, and types of conflict across taxa can inform attitudes and beliefs around each target species that principally dictates management attention (Figure 4; Box 1).

**Case studies: Coyotes versus brown rats**

When considering the consequences of conflict for urban wildlife populations, perhaps no two species are more representative than coyotes and rats. These two species are unique among wildlife species because they have expanded their geographic ranges, while most others have become more restricted (Puckett et al., 2016; Thurber & Peterson, 1991). The ecological success of coyotes and rats is most likely due to their broad habitat and dietary niches (Gehrt & Riley, 2010; Guiry & Buckley, 2018), and high behavioral flexibility and tolerance for human disturbance (Breck et al., 2019; Feng & Himsworth, 2014; Murray & St. Clair, 2015; Schell et al., 2018; Young, Hammill, et al., 2019). However, the success of coyotes and rats has led to high rates of conflict in cities throughout their respective ranges. While both species come into conflict with people for various reasons, coyotes are uniquely feared for rare but alarming physical attacks on people and domestic animals (White & Gehrt, 2009) and conflicts are disproportionately caused by so‐called “problem individuals,” which exhibit unusually high levels of habituation to human presence (Schmidt & Timm, 2007). Conversely, rats cause over 20 billion USD in property damage annually by chewing infrastructure and spoiling food stores (Pimentel et al., 2005) and transmit many zoonotic pathogens (Himsworth et al., 2013). Due to these differences, coyotes are often managed at the individual level by hazing or removing problem individuals (Breck et al., 2017), while the goal of rat management is to reduce densities via trapping or poisoning (Combs et al., 2019). These approaches may have important consequences for evolutionary change in cities. For coyotes, nonlethal management strategies such as hazing may select for plastic phenotypes, while the removal of problem individuals may select for less bold phenotypes. For rats, population‐level culling to reduce rat densities may impose less selection than directly targeting individuals exhibiting atypical behaviors. However, intense lethal management will undoubtedly impose a selective pressure favoring neophobia and resistance to poisons, both of which have been documented in detail (Desvars‐Larrive et al., 2017; Feng & Himsworth, 2014). Changing management practices toward both species will serve as natural experiments for urban evolution. For example, nonlethal management of urban coyotes is often recommended for concerned urbanites (Young, Draper, et al., 2019; Young, Hammill, et al., 2019) and rodenticides are now restricted in some jurisdictions (Quinn et al., 2019). Incorporating evolutionary concepts in such management decisions will help inform successful mitigation strategies.

### 3.1 Socioeconomic drivers of conflict

The unequal distribution of capital and income greatly contributes to the distribution of wildlife, as well as the relative proportion of native to introduced species (Leong et al., 2018; Schell et al., 2020; Warren et al., 2013). The luxury effect suggests that neighborhood wealth influences emergent patterns of urban biodiversity and community structure (Grove et al., 2014; Hope et al., 2003; Leong et al., 2018), and though wealth–biodiversity relationships are not universally positive (Gerrish & Watkins, 2018; Kuras et al., 2020; Watkins & Gerrish, 2018), repeated evidence across the globe has supported this hypothesis (Chamberlain et al., 2020). Fewer studies have investigated whether economic inequality shapes beliefs and attitudes toward wildlife in urban environments. However, recent research suggests that individuals with wealth from developed countries tend to have more favorable views of wildlife due to greater frequencies of positive interactions (Soga & Gaston, 2020). Whether these trends hold true for developing urban centers, particularly across the global south, is uncertain.

The distribution of and access to green spaces is significantly reduced for low‐income communities relative to wealthier communities in cities (Rigolon et al., 2018; Wolch et al., 2014). Reductions in vegetation cover and green space, compounded with other environmental disturbances (e.g., pollutants human densities, urban heat island effects), necessarily constrain available niche space for certain wildlife in favor of non‐native and pest species in low‐income neighborhoods (Leong et al., 2018). For instance, reductions in vegetation cover and plant biodiversity in low‐income neighborhoods (Schwarz et al., 2015) often covary with greater pest species abundances (e.g., brown rats, *Rattus norvegicus;* mosquitoes, *Aedes aegypti*) that frequently cause property damage and represent significant disease vectors, disproportionately increasing risks of zoonotic disease transmission for low‐income residents (Byers et al., 2019; Mathanga et al., 2016; Murray, Fidino, et al., 2020; Peterson et al., 2020). As a result, luxury effects may indirectly determine the types of human–wildlife interactions experienced by different socioeconomic groups. Centering environmental justice in improving green space access, quality, and equity may subsequently drive positive attitudes with wildlife by providing positive interactions with nature, which can bolster overall support for wildlife‐friendly policies in cities.

### 3.2 Religion, culture, and media

How religious traditions view the environment and wildlife can shape how people respond to emergent conflicts from individual organisms (Dickman et al., 2013; Manfredo & Dayer, 2004). For instance, rhesus macaques (*Macaca mulatta*) in Dehradun, India, are commonly involved in property damage and injury to humans, but are also revered in Hinduism, which results in ambivalent attitudes toward conflict management by members of the public (Anand et al., 2018; Beisner et al., 2015; Saraswat et al., 2015). Ritualized feeding in Delhi, India, of black kites (*Milvus migrans*) by citizens combined with the city's inefficient waste removal is linked to higher recorded attacks and aggression on humans, yet the affected human communities demonstrate heightened empathy and tolerance for the kites (Kumar et al., 2018, 2019). Further, residents of Jodhpur, Rajasthan, India, feed urban Hanuman langurs (*Semnopithecus entellus*) in reverence to the monkey god, Hanuman (Waite et al., 2007), whereas tourists report hostile and agonistic interactions as a residual effect of habituated monkeys (Sharma et al., 2010).

The influence of sociocultural conditions can exaggerate hostilities toward specific taxa regardless of the actual risk of conflict (Peterson et al., 2010). For example, individual attitudes and beliefs toward coyotes in urban and suburban regions of Denver strongly predict support for lethal control measures over nonlethal strategies such as hazing and education (Draheim et al., 2019). Conversely, growing interest in wildlife as pets can be influenced by popular culture trends. For instance, the global popularity of the Harry Potter movie franchise led to an increase in demand for owls as pets, with a noticeable impact on the wildlife trade (Nijman & Nekaris, 2017). In both examples, culturally informed views on specific wildlife can negatively impact wild population dynamics and lead to novel species interactions that have the potential to increase pathogen transmission risks.

How news and social media portray human–wildlife conflict can also play a substantial role in how certain species are perceived (Nyhus, 2016). For example, recent media reporting has fueled animosity toward bats due to the COVID‐19 pandemic, despite repeated evidence emphasizing that human activities are the primary predictors for our current public health crisis (MacFarlane & Rocha, 2020). Similarly, negative media on urban leopards in Mumbai, India, can exacerbate negative stereotypes, which require targeted awareness campaigns, education, and multimedia approaches to alter negative beliefs (Hathaway et al., 2017). Media awareness workshops in Mumbai, India, for example, have worked to combat negative views around urban leopards as aggressors while promoting behaviors that help prevent human‐leopard conflicts (Bhatia et al., 2013; Hathaway et al., 2017). Some have additionally suggested that leopards have indirect public health benefits by hunting feral dogs, which consequently reduces dog bites in the city (Braczkowski et al., 2018).

## 4 MANAGEMENT‐INDUCED PHENOTYPIC AND GENOTYPIC CHANGE

Management decisions to resolve conflict act as a selective agent by either (a) removing individuals from a population; (b) controlling overall growth of a population; or (c) targeting behaviors and traits that incite conflict (Box 1). The varied techniques and goals of wildlife management work at different ecological and geographic scales, and as a result, have varying consequences for organismal evolution in cities. In addition, wildlife adaptations to management decisions may produce significant feedback (Honda et al., 2018), driving coevolution between humans and wildlife in cities (Jørgensen et al., 2019; Marzluff & Angell, 2005; Mysterud, 2010). Moreover, wildlife adaptations to management decisions may produce directional, stabilizing, or disruptive selection for phenotypic traits (e.g., boldness) that drive mean‐level population differences across cities (Figure 2).

Determining the proper management strategy is nontrivial, because these decisions may elicit adaptive wildlife responses that negate the long‐term efficacy of the management action (Swan et al., 2017). Understanding how differences in lethal and nonlethal management actions affect the emergence of novel traits and the strength of selection across urban taxa is essential to creating robust and dynamic management (Figure 3). What constitutes an urban area and the extraordinary variability in urban metrics across developed and developing cities (Moll et al., 2018, 2020) requires markedly distinct management solutions. Further, acknowledging how the frequency and severity of conflict—driven by social perceptions of wildlife—dictate the intensity of management action helps to predict the potential evolutionary outcomes of wildlife management efforts (Figure 4).

### 4.1 Lethal management: Targeted removals

Selective removal of targeted animals is arguably the strongest and most consistent form of management‐driven directional selection for urban wildlife (Hendry et al., 2017; Nyhus, 2016). Individuals with specific behavioral phenotypes that are conflict‐prone are selectively removed from the population to avoid conflict escalation. As a result, we may expect that urban environments with stronger and more consistent targeted removal programs should exhibit greater selective costs for bold or aggressive individuals (Swan et al., 2017). For instance, lethal removal of conflict‐prone individuals has been suggested as a strategy to manage urban deer (Honda et al., 2018); however, because boldness is a phenotype derived from genetic and environmental interactions, it is possible that culled individuals will be replaced by the next boldest individuals in a population (Found & St. Clair, 2019). Removal of individuals to control population size may also exacerbate patterns of increased genetic drift and decreased genetic diversity already experienced by urban populations (Combs et al., 2018; Edelhoff et al., 2020; Miles et al., 2019).

### 4.2 Lethal management: Rodenticides

The most notable example of genetic change in response to lethal management may be evolved resistance to anticoagulant rodenticides in urban rats (Haniza et al., 2015). Integrated pest management has widely utilized anticoagulant rodenticides to control rats since the introduction of warfarin as a rodenticide in 1948 (Desvars‐Larrive et al., 2017). The initial efficacy of such practices led to rodenticide products readily available for homeowners and individual residents to use at their leisure. Within a decade, individual rats expressed resistance to warfarin via genetic mutations (Boyle, 1960). In the following years, the intense use of anticoagulants created a strong selection pressure that increased the prevalence of resistant rats in many cities. To counteract this diminished effectiveness, "second‐generation" anticoagulant rodenticides were developed; however, rat populations have evolved resistance to these compounds as well (Desvars‐Larrive et al., 2017). Similar evolved resistance appears in mosquitos (*Culex pipiens*) and bedbugs (*Cimex lectularius*) in response to select pesticides (Asgharian et al., 2015; Romero & Anderson, 2016). Currently, the application of rodenticides and pesticides are geographically and temporally acute, determined by need and severity of pest conflict. As a result, these toxicants create heterogeneous fitness landscapes that can result in genetic bottlenecks (nonadaptive change) and selection for toxicant resistance (adaptive) mutations.

Bioaccumulation of these rodenticides can result in unintentional secondary poisoning of nontarget species at higher trophic levels in urban systems (Elliott et al., 2016; Murray et al., 2019; Riley et al., 2007; Serieys et al., 2015, 2018). The long‐term persistence of second‐generation anticoagulant rodenticides (SGARs) in animal tissues increase exposure risks for secondary and tertiary predators that ingest rodent carcasses or incapacitated rodents that have ingested SGARs (López‐Perea & Mateo, 2018). For example, recent evidence from urban bobcats (*Lynx rufus*) in Los Angeles suggests SGARs in blood and liver tissues increase with urban land use (Serieys et al., 2015), promote immune dysfunction (Serieys et al., 2018), and impact differential gene expression of immune‐related genes (Fraser et al., 2018). Increasing exposure to rodenticides with increasing urbanization has similarly been documented for mountain lions (*Puma concolor*) and coyotes (Poessel et al., 2015; Riley et al., 2007). Hence, rodenticides have broad fitness outcomes that extend far beyond the target species.

### 4.3 Nonlethal control

Developing nonlethal deterrents that are successful long‐term is a major challenge due to difficulty of deployment, enhanced learning, and selection for behavioral plasticity, with the latter two leading to cognitive arms races and coevolution between humans and wildlife (Barrett et al., 2019; Marzluff & Angell, 2005). Visual, audio, taste, or scent aversion strategies yield mixed results and can be difficult to employ. For example, the use of predator scent as a repellent has shown promise in deterring unhabituated eastern gray kangaroos (*Macropus giganteus*), but implementation poses challenges for managers (Descovich et al., 2016). A variety of taxa have demonstrated habituation to nonlethal deterrents, such as effigies and frightening devices, rendering such management efforts ineffective when applied alone (VerCauteren et al., 2010). Greater exposure to humans and anthropogenic structures without selective cost also contributes to increasing urban wildlife boldness (Figure 2), as evidenced by decreased flight initiation distances when approached by humans (Breck et al., 2019; Uchida et al., 2016) and approach time toward novelty (Greggor et al., 2016; Jarjour et al., 2019). In addition, individual variation in physiology and life history traits can compound with cognition and behavioral traits to hinder the success of certain nonlethal deterrents (Barrett et al., 2019).

Habitat modification also serves to mitigate human–wildlife conflict. For example, physical barriers, such as fences, are employed to separate terrestrial wildlife from areas of human development. The application of spikes, coils, nets, and monofilament wires to surfaces is usually successful in deterring undesired feeding and roosting by birds when applied correctly (VerCauteren et al., 2010). Managers may also remove water sources, secure food subsidies, or alter vegetative composition to make particular conflict zones less appealing to wildlife (VerCauteren et al., 2010), which further reduces potential ecological and evolutionary traps that jeopardize wildlife fitness (Greggor et al., 2019; Lamb et al., 2017). Although fences present some benefits for wildlife conservation, they often result in unintended, negative consequences (Woodroffe et al., 2014). Fences have been shown to cause injury and reduce landscape connectivity, disrupting daily activity and migration of terrestrial mammals (Jakes et al., 2018). In addition, fencing and other anthropogenic barriers constrain wildlife access to essential habitats, reduce animal movement, and contribute to moderate losses in genetic diversity (Osipova et al., 2018).

Translocation is a popular nonlethal management strategy that has recently increased in implementation (Germano et al., 2015). This may be due to public views and beliefs that this strategy is a humane alternative to targeted removal or pesticides and is less intensive than repeated behavioral deterrents. However, the efficacy of this strategy is seldom clear and postrelease survival is generally poor (Fontúrbel & Simonetti, 2011; Germano et al., 2015; Lehrer et al., 2016; Massei et al., 2010). Human‐related mortality (e.g., vehicle collisions, hunting) accounts for approximately 80% of carnivore deaths after a translocation event (Fontúrbel & Simonetti, 2011). It is common for problem individuals to widely disperse or return to their point of origin after translocation (i.e., “homing”), making their initial removal ineffective (Fontúrbel & Simonetti, 2011). Urban individuals that survive and do not return to their original location may be susceptible to predation (Lehrer et al., 2016) or exhibit problem behaviors in their relocated environment (Athreya et al., 2011). In the few cases where urban translocation has been successful (Nelson & Theimer, 2012), the sweeping removal of entire family groups creates genetic bottlenecks that fundamentally shape urban population genetic structure (Weeks et al., 2011).

## 5 APPLICATIONS FOR ADAPTIVE WILDLIFE MANAGEMENT

**FIGURE 5:**
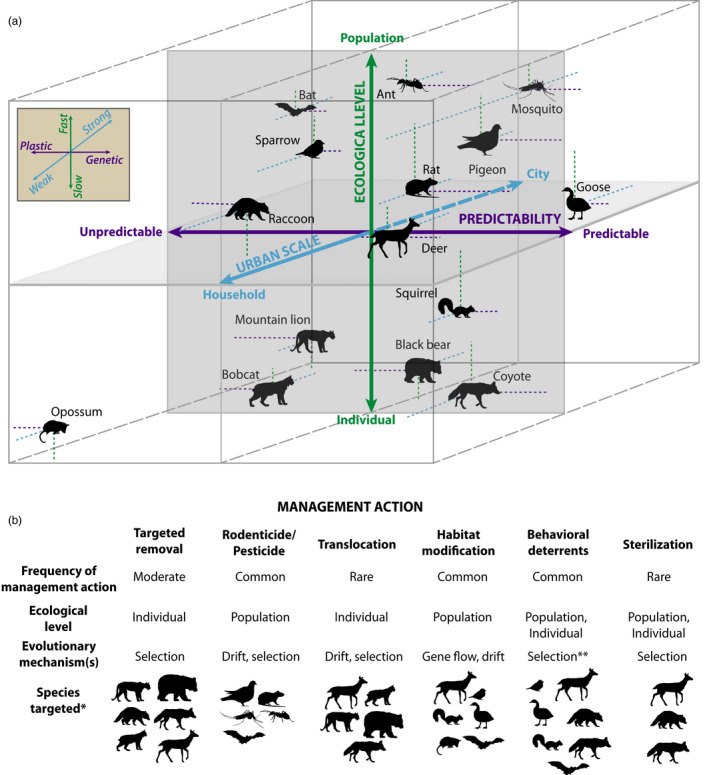
A conceptual model and heuristic model predicting the strength, rate, and type of phenotypic change (i.e., plastic or genetic) due to management action scale, predictability, and ecological level. (a) The scale of management application, how consistent management actions are, and the overarching goal (i.e., individual problem animal removal vs. broad‐scale population control) differentially affect evolutionary change across urban taxa. (b) Specific management actions have varying levels of implementation, operate at different ecological levels, and influence different adaptive (i.e., selection) and nonadaptive (i.e., drift, gene flow) evolutionary mechanisms. The species targeted also vary with respect to the management action taken. **Behavioral deterrents are a special case of selection, as aversive conditioning may lead to social learning or transgenerational plasticity that ultimately leads to variance in selection but is inherently not targeting specific gene frequencies

Wildlife managers and practitioners inherently value evolutionary principles and their relevance to wildlife management efforts (Cook & Sgrò, 2018). Time and budget constraints paired with the near‐immediate call for management action from the public, however, place a distinct burden on managers to quickly develop effective strategies. Clearly articulating the links between urban evolution and wildlife management, with succinct recommendations and potential outcomes, is necessary for effective communication across these disciplines. The spatial extent, ecological level, and predictability of wildlife management implementation are intrinsically linked to the strength and rate of evolutionary change (Figure 5). Further, phenotypic signatures of urbanization are trophic‐ and scale‐dependent (Strubbe et al., 2020), and scalar differences within and across cities are fundamentally driven by social determinants of urban landscapes (Liu et al., 2007; Zipperer et al., 2011), making it difficult to implement broad management recommendations.

Discerning whether observed changes in urban traits are plastic or genetic is not only an essential question in urban evolutionary ecology (Alberti et al., 2017; Donihue & Lambert, 2014; Ouyang et al., 2018; Rivkin et al., 2019; Schell, 2018), but also informs the most effective management and conservation strategy (Lambert & Donihue, 2020). For instance, if expressions of boldness are predominantly plastic or learned, deterrents could effectively be used to instill fear dynamics and promote cautionary behavior without lethal removal (Clucas & Marzluff, 2012). Associative learning through aversive conditioning could also bolster population‐level fear, even if certain individuals have never encountered negative anthropogenic stimuli (Barrett et al., 2019). If the trait is principally genetic, then improved identification and targeted removal of repeat problem animals may functionally reduce problem‐associated alleles in the population (Swan et al., 2017).

Strategies to mitigate human–wildlife conflict would ideally be implemented early in the development of urban areas and would accommodate changes in patterns of conflict that may arise during development. For example, Khan et al. (2018) documented increased conflicts with leopards in developing areas of Pakistan; such knowledge of how species respond to developing areas could be used in urban planning. Understanding species responses to urbanization (Moll et al., 2020; Santini et al., 2019), subsequent potential conflict patterns (Goswami et al., 2015), and the evolutionary impacts (Rivkin et al., 2019) could prevent the development of maladaptive behavior in wildlife species and help urban landscape planners minimize conflicts during development (Nilon et al., 2017). In fact, there is a growing interest in smart growth to lessen environmental impacts of urban development (Theobald et al., 2005). Studies of wildlife behavior and human–wildlife conflicts along the urban–rural interface, combined with modeled projections of future human development (Yovovich et al., 2020), may provide insight into how or whether management strategies should shift with urbanization; for example, cougars expand their niche along with urban expansion (Moss et al., 2016), alter prey selection (Smith et al., 2016), and shift habitat use (Maletzke et al., 2017; Yovovich et al., 2020) based on human development characteristics.

Understanding how natural and built structures coalesce to form heterogeneous fitness landscapes is critical to diagnosing conflict zones, informing which habitat modifications may yield the most positive results for conflict mitigation (Nyhus, 2016). For instance, the spatiotemporal concentration of natural or artificial food subsidies may create ecological and evolutionary traps for wildlife (Lamb et al., 2017; Lewis et al., 2015). Deterring maladaptive resource use in human‐dominated environments may require several nonlethal strategies that appropriate cognitive mechanisms (Greggor et al., 2019). Involving urban planning and policymakers can also help to develop built structures that promote connectivity and increase gene flow, combating against urban‐driven loss in genetic diversity and human damages arising from collisions on roads (Schmidt et al., 2020). Green infrastructure in cities, including green roofs, wetlands, and wildlife corridors, provides valuable passages, stepping stones, and refuges for wildlife to avoid several types of conflicts with people (Lundholm, 2015). Comprehensive implementation of green infrastructure is an effective tool in mitigating human–wildlife conflict (Ravenelle & Nyhus, 2017), and examples such as smooth‐coated otter (*Lutrogale perspicillata*) conservation in the nation city of Singapore provide a blueprint. Sustained urban greening and public communication created refugia for otters while simultaneously bolstered social views on the value of the species (Theng & Sivasothi, 2016). Hence, striking a balance between wildlife tolerance of cities while reducing potential conflict will require a similar nuanced and targeted approach.

## 6 CONCLUSION

Our world is becoming increasingly urbanized, compelling organisms to adjust under rapid timescales. Such adjustments are exacerbating levels of conflict globally, with the recent global COVID‐19 pandemic a significant case study. The convergence of human and wildlife populations in urban areas has substantial feedbacks on regional and international economies, conservation efforts, and public health initiatives. Our changing relationships with urban wildlife are affecting how we view, conserve, and manage wildlife, all of which will dictate our success in promoting coexistence. Hence, diagnosing how conflicts arise and change over time is a priority for public health, the environment, and society. It is imperative that evolutionary biologists work with urban planners, wildlife practitioners, social scientists, and policymakers create holistic efforts leveraging the strengths of our communities to benefit all organisms in an increasingly urbanizing world.

## CONFLICT OF INTEREST

None declared.

## Data Availability

There are no data associated with this article.
